# Soil microbial communities are more disrupted by extreme drought than by gradual climate shifts under different land-use intensities

**DOI:** 10.3389/fmicb.2025.1649443

**Published:** 2025-08-07

**Authors:** Lena Philipp, Evgenia Blagodatskaya, Mika Tarkka, Thomas Reitz

**Affiliations:** ^1^Department of Soil Ecology, Helmholtz-Centre for Environmental Research, Halle, Germany; ^2^German Centre for Integrative Biodiversity Research (iDiv), Halle-Jena-Leipzig, Leipzig, Germany; ^3^Institute of Agricultural and Nutritional Sciences - Crop Research Unit, Martin Luther University Halle-Wittenberg (MLU), Halle, Germany

**Keywords:** global change, drought, cropland, grassland, microbial functions, microbial networks

## Abstract

**Introduction:**

Extreme events like droughts are expected to increase in frequency due to climate change and will affect ecosystems and their associated key functional components particularly soil microbial communities. Studies simultaneously addressing a range of climate stressors, such as extreme drought events and gradual long-term shifts in precipitation and temperature on soil microbial diversity, community composition and function in agricultural systems are limited.

**Methods:**

Here, we present a data set from a field site in Central Germany comprising two spring growing seasons, one with normal precipitation amount, the other experiencing an extreme drought. Further, the experiment included a climate treatment simulating climate change induced gradual shifts in precipitation and temperature in croplands and grasslands under varying management intensities.

**Results and discussion:**

Our findings demonstrate that the extreme drought had a stronger effect on microbial biomass, functions and community composition than the mild experimental climate treatment mediated by soil moisture differences. The fungal communities were more responsive to the drought than the bacterial community, particularly in croplands, where we observed higher C-cycling enzymatic activities under drought. In contrast, microbial functions in grasslands remained largely unchanged in grasslands under drought, implying lower sensitivity to drought in grassland than cropland systems. However, intensively managed grasslands appeared less stable in community composition and function than extensively managed grasslands, which was also observed in constructed co-occurrence networks. Overall, our results suggest that intensively managed systems are more vulnerable to extreme drought conditions with an increase of fungi with pathogenic potential which may further destabilize soil microbial communities in the future These findings underscore the need to consider multiple stressors such as extreme events and land-use intensity in order to understand the soil microbial response to global change.

## 1 Introduction

In terrestrial ecosystems, soil microbial communities play a crucial role in maintaining ecosystem functioning, resilience, and stability (Hartmann and Six, 2023). In agroecosystems in particular, they are essential for processes such as nutrient cycling, organic matter decomposition, nitrogen fixation and carbon storage (Falkowski et al., 2008; Kallenbach et al., 2016). Additionally, microbes support plants by facilitating nutrient uptake, producing plant growth-promoting hormones, and protecting against pathogens, through the production of antimicrobial compounds or competitive exclusion (Compant et al., 2019; Eichmann et al., 2021). These functions are essential for maintaining soil fertility and plant productivity, as well as for ensuring ecosystem resilience to differing environmental conditions. Microbial communities with high functional diversity and redundancy can buffer environmental changes and maintain functionality even under disturbance and stress (Dubey et al., 2019; Martins et al., 2024). One important but underexplored aspect is the interaction of bacterial and fungal communities. Despite their interdependent roles in the decomposition process (de Menezes et al., 2017), those are often studied separately. Fungi can transport nutrients to the vicinity of prokaryotic communities via their mycelia (Nazir et al., 2010) and fungal-endobacterial symbiosis can prevent pathogen infection (Büttner et al., 2021). Therefore, the interplay of bacterial and fungal communities is likely to affect ecosystem functioning (Frey-Klett et al., 2011) and it is necessary to study both soil prokaryotic and fungal communities to adequately estimate soil functionality in response to the climatic and anthropogenic impact (Wagg et al., 2019).

Climate change and land-use intensification directly influence soil microbial habitats, by altering temperature, water and nutrient availability and disturbance frequency, with consequences also for microbial functions. In Central Europe, precipitation levels will not decrease but patterns will shift between seasons and precipitation variability will increase (Pendergrass et al., 2017). This results in a higher frequency of single events such as extreme droughts (Spinoni et al., 2018; Goffin et al., 2024), while accumulated climate change effects over the long-term, induced by average temperature increase and changed precipitation patterns, have to be considered as well. However, studies that simultaneously investigate both factors (extreme events and long-term changes) are rare, even though both factors influence soil moisture, which is a key driver of nutrient availability, microbial activity and ultimately microbial functions. Higher water availability can promote nutrient release and stimulates microbial activity and growth (Osburn et al., 2024). In contrast, drought conditions can reduce resource availability, leading to microbial dormancy or inactivity (Lennon and Jones, 2011). Drought may also indirectly affect soil microbial communities through changes in plant communities. For instance, drought during the growing season can strongly reduce plant productivity, which in turn can limit soil nutrient cycling, potentially affecting plant–soil processes (Muhammad et al., 2025), depending on the timing of the drought within the growing season (Xiao et al., 2023). Drought can affect the abundance of specific microbial taxa and functional groups, and thereby alter functional parameters (Schimel, 2018), while multifunctionality is maintained (Canarini et al., 2021). In response to drought, microbial communities may adapt to drought by selecting for species with a competitive advantage under such conditions. Drought reportedly affects fungal and bacterial communities differently, e.g., fungi tend to be more drought resistant than bacteria (Barnard et al., 2013; Dacal et al., 2022), but other studies report contrasting results, suggesting that drought responses are context dependent (Kaisermann et al., 2015; Maisnam et al., 2023). Moreover, it remains unclear to which extend the drought response depends on agricultural management practices (Azarbad, 2022). Given these uncertainties, more research is needed to study interacting effects of drought and land use on soil microbial communities.

Grasslands and croplands provide contrasting examples of how different management regimes shape microbial communities. Grasslands, characterized by relatively undisturbed and diverse vegetation, improve soil structure and porosity through deep and fibrous root systems, enrich organic matter, improve soil fertility and water-holding capacity, store carbon in roots and soil (Liu et al., 2020; Panchal et al., 2022; Kuka and Joschko, 2024). Microbial diversity and activity are promoted through decomposition of diverse plant material. In contrast, croplands, subject to intensive agricultural practices including regular habitat disruption due to tillage, exhibit lower functional and structural diversity of soil microorganisms (Banerjee et al., 2024; Peng et al., 2024). In addition, differences in management intensity, e.g., through fertilization between conventional and organic croplands or intensively and extensively managed grasslands can drastically affect the diversity of soil microbial communities (Tsiafouli et al., 2015; Gossner et al., 2016). These fundamental differences underscore the importance of identifying the microbial processes that promote ecosystem functioning and stability across different land-use types and management intensities. Comparative studies that include multiple management regimes, ranging from intensive farming management to low intensity grassland management are rare in the context of climate change research. Our study site—the Global Change Experimental Facility (GCEF)—provides a unique experiment where we can study five land-use types under ambient climate and under a future climate scenario. Utilizing this experimental design, we analyzed a two-year dataset that captured contrasting environmental conditions during the growing season: one year characterized by normal spring precipitation, and another marked by a dry spring, resulting in a drought event. This combination enabled a simultaneous evaluation of two aspects of climate change: (i) the cumulative effects of 8 years of experimental climate manipulation, and (ii) the impact of climate extremes (Supplementary Figure S1). This approach provides a more nuanced understanding of how gradual climate shifts and extreme events interact to influence soil microbial communities. By integrating eco-physiological indicators and amplicon sequencing of bacterial and fungal communities, this work aims to link both structural and functional diversity patterns of microbial communities across land-use types under climate change. Specifically, we investigated how microbial communities respond to an extreme dry compared to a normal spring season, focusing on effects at peak biomass production across agroecosystem management types.

We hypothesize that (i) the magnitude of the experimental future climate effects on microbial community structure and functions will vary between a dry and a normal spring season, due to soil moisture thresholds and differently abundant microbial taxa. We expect that the effects will be more pronounced in a normal than in a dry spring season, since higher precipitation under a future climate will enhance soil water availability, thereby stimulating microbial activity and nutrient cycling. In contrast, under dry conditions crucial soil moisture thresholds even under future climate conditions are not reached. Second, we hypothesize that (ii) grassland microbial communities will be more resilient to changes induced by experimental future climate and extreme events, due to less frequent management disturbances compared to croplands. Further, a higher functional redundancy of microbial taxa will allow them to maintain biomass, activity and functional potential under changed environmental conditions. Third, we hypothesize that (iii) the effects of experimental climate change and extreme events on individual taxa will be more pronounced in intensively managed than in extensively managed systems. As land-use intensification (conventional vs. organic farming and intensive meadow vs. extensive grasslands) will promote decomposer dominated communities and lower microbial network complexity through fertilization effects, we expect lower stability in intensively managed systems toward changed environmental conditions.

## 2 Methods

### 2.1 Study site

The Global Change Experimental Facility (GCEF) in Bad Lauchstädt, Central Germany (51°23′35′′N, 11°52′55′′E, 118 m a.s.l., mean annual temperature of 9.0°C and a mean annual precipitation of 483 mm between 1896 and 2021), is a field experiment, established in 2013. The soil at the study site is a Haplic Chernozem. It comprises five land-use types (conventional and organic farming, intensive and extensive meadow, extensive pasture) which are subjected to the ambient current climate and a future climate treatment including a daily mean average temperature increase of 0.55°C and a change in precipitation regime (+10% in spring and autumn, −20% in summer compared to the ambient control) in a full factorial design. The mobile roof and side panel construction on future climate plots is closed every night to accomplish passive warming of the plots and passive exclusion of precipitation. Furthermore, an irrigation system was installed to adjust the precipitation amount to the seasonally different precipitation regime compared to the ambient control plots if necessary (see Schädler et al., 2019 for further details).

### 2.2 Soil sampling

Soil samples were taken in May 2022 and 2023 from all plots as a composite of three cores (diameter: 1.5 cm) in the depth 0–15 cm, sieved (2 mm), and stored at 4°C for analysis of soil moisture, microbial biomass, respiration and enzymatic activities or −20°C for analysis of available nutrients and air-dried for analysis of pH and TC and TN. Samples for Illumina MiSeq Amplicon Sequencing were stored at −80°C. Precipitation in the spring season (starting from March 1) prior to sampling were 25 mm in 2022 and 81 mm in 2023. The mean precipitation from 1997 to 2021 in the same time frame of the spring season was 66 mm (Gründling et al., 2022; Schädler and Remmler, 2024).

### 2.3 Soil abiotic parameters

Gravimetric soil moisture was determined in a halogen moisture analyzer (Kern DBS60-3, Kern & Sohn GmbH, Germany). TC and TN were determined in an CHN elemental analyzer (Vario EL III, Elementar Analysensysteme GmbH, Langenselbold, Germany). Mineral nitrogen (ammonium and nitrate fraction) was extracted from fresh soil with 1 M KCl (1:4 w/v, 1.5 h) and filtered (Whatman Schleicher and Schuell 595 1/5 Ø 270 mm filter). Concentrations of extracted NH_4_^+^-N and NO_3_^−^-N were determined in a flow injection analyzer (FIAstar 5000, Foss GmbH, Rellingen, Germany). Plant available P and K were extracted with the double lactate method (1:50 w/v, pH 3.6, 1.5 h), P concentration was determined colorimetrical with the molybdenum blue method. K concentration was determined with an ion-selective electrode (Mettler Toledo SevenExcellence pH/Ion meter, Gießen, Germany). pH was measured with a pH electrode (Mettler SevenEasyse pH meter, Gießen, Germany) after air-dried soil was suspended in 0.01 M calcium chloride solution (1:2.5 w/v, 1 h).

### 2.4 Microbial biomass

Microbial carbon and nitrogen were determined using the chloroform fumigation extraction method. Chloroform-fumigated and non-fumigated soil samples were extracted with 0.05 M K_2_SO_4_ solution (1:4 w/v, 30 min). Extracts were centrifuged (10 min) and C and N concentrations were determined in a flow injection analyzer (FIAstar 5000, Foss GmbH, Rellingen, Germany). To account for the non-extractable part of microbial C and N, correction factors (k_EC_ = 0.45 for C and k_EN_ = 0.54 for N) proposed by Joergensen (1996) and Joergensen and Mueller (1996) were used to calculate total microbial C and N.

### 2.5 Soil biological parameters

Basal respiration was determined in a RESPICOND V respirometer (Respicond AB, Roberstfors, Sweden) using 30 g of fresh soil, incubated at 22°C. Respired CO_2_ was captured in KOH (10 mL, 0.6 M). The basal respiration rate was calculated from the change in conductance of the KOH solution measured every 20 min. CO_2_ production rate was calculated in mg respired C-CO_2_ g^−1^ dry soil h^−1^. After 48 h, microbial growth was induced through addition of a glucose-nutrient solution. Respiration rate was measured for another 48 h. Enzymatic activity potentials were determined using a fluorometric method (4-methylumbelliferone (MUF)-fluorescence). Briefly, the assay was conducted under substrate saturating conditions (assay volume: 250 μL). Fresh soil samples were solubilized and homogenized in 50 mM sodium acetate buffer (pH 5) in an ultrasonic bath for 5 min. MUF-linked substrates (300 μM) specific for each enzyme (cellulase, xylosidase, β-glucosidase, acid phosphatase, N-acetyl-glucosaminidase (NAG) and sulfatase) were incubated with soil suspension at 25°C. MUF standards (1.25 and 2.5 μM) with and without soil suspension were incubated in parallel for each soil sample to account for sample specific quenching effects. Enzymatic reactions were stopped after 1 h upon addition of 30 μL NaOH (1 M). MUF fluorescence (ex/em: 360 nm/465 nm) was measured in an Infinite 200 PRO instrument (Tecan Group Ltd., Männedorf, Switzerland).

### 2.6 Amplicon sequencing of 16S-rRNA-gene and ITS2-region

DNA was extracted from soil using the Qiagen DNeasy PowerSoil Pro Kit. 16S-rDNA-gene and ITS2-region fragments were amplified in a PCR using primers (515f: GTGYCAGCMGCCGCGGTAA, 806r: GGACTACHVGGGTWTCTAAT for16S rDNA and ITS4: TCCTCCGCTTATTGATATGC and fITS7: GTGARTCATCGAATCTTTG for ITS2 region) and the KAPA HiFi DNA polymerase and 40 ng of extracted DNA per PCR reaction. PCRs were conducted in triplicates (cycler program 16S: 95°C 3 min, (98°C 20 s, 55°C 20 s, 72°C 15 s) x25, 72°C 5 min, cycler program ITS2: 95°C 3 min, (98°C 20s, 56°C 20 s, 72°C 20s) x30, 72°C 5 min). Success of PCR was checked with agarose gel electrophoresis and triplicate PCR products were pooled and purified with AmpPure XP Beads. PCR products were indexed with Illumina MisSeq adapters (Illumina Nextera XT index primers) in an additional PCR and purified with AmpPure XP Beads. Concentration of the indexed PCR products was measured with a Nanodrop ND-8000 spectrophotometer. DNA was equimolarly pooled and sequenced in pair-end sequencing with an Illumina MiSeq device in two sequencing runs. Sequencing reads were processed using the dadasnake pipeline (version 11) including quality filtering, primer and chimera removal, ASV and taxonomy assignment and functional predictions (Weißbecker et al., 2020). For taxonomy assignment of prokaryotes SILVA138 SSU database was used for fungi UNITEv10 database was used. Additionally, functional traits of fungi were assigned with FungalTraits. Finally, sequences originating from chloroplast and mitochondrial DNA were removed. A total of 51,627 prokaryotic ASVs and 5,930 fungal ASVs entered further analysis.

### 2.7 Statistical analysis

All statistical analyses were conducted in R (version 4.2.2). Soil and microbial parameters were analyzed using linear-mixed effect models, with climate, land use, year and their interaction as fixed effects, while subplot nested within mainplot was included as a random effect to account for split-plot design and repeated measurements. Model significance was tested with a three-way ANOVA. Enzyme activity potentials were normalized to microbial biomass C prior to statistical analysis. Microbial growth parameters were estimated from substrate induced respiration curves using the twKinresp package (Wutzler et al., 2012). Microbial metabolic quotient qCO_2_ was calculated as ratio of microbial C and basal respiration. For microbial community analysis, taxa with prevalence less than 10% of all samples were removed prior to analysis. Differences in microbial community composition were analyzed with PERMANOVA on the Bray–Curtis dissimilarity. For each categorial variable (climate, land use and year) a separate PERMANOVA (nperm = 9,999) was conducted to account for the split-plot design and to control for the factor level and correct calculation of *F* value and *p* value using the permute package. Differential abundance analysis of microbial taxa between land-use types at the genus level was done using the ANCOMBC2 method (Lin and Peddada, 2024), including pairwise comparisons between each land-use type per year (significance level = 0.05). FungalTraits-derived primary fungal lifestyles were subjected to differential abundance analysis with ANCOMBC2 as well. Microbial co-occurrence networks were constructed separately for each land-use type per year (*n* = 20) using the SpiecEasi package, with taxa grouped at the family level (parameter call spiec.easi: method = mb, sel.criterion = bstars, nlambda = 40, rep.num = 99, threshold = 0.05). Association matrices were derived from SpiecEasi constructed networks and pairwise network statistics were computed with the NetCoMi package in R. Clusters were identified with the cluster_fast_greedy function. Hub nodes (indicating key stone taxa) were defined above 0.95 quantile of degree and closeness centrality. That and difference in other network properties of each pairwise land-use combination were determined using Jaccard similarity index. Structural equation models were constructed using the piecewiseSEM package. PCoA scores of Bray–Curtis dissimilarity were used as indicators of bacterial and fungal community composition (PCoA axis 1). All measured variables were z-transformed prior to SEM construction, an activity index was calculated as the average of all z transformed enzymatic activities. A network complexity score was calculated as the average of the z-transformed edge numbers, z-transformed and inversed modularity and z-transformed and inversed average path length. Each individual model formula included the term LANDUSE + CLIMATE + YEAR as fixed factors and (1|MAINPLOT) as a random factor to account for the split-plot-design of the GCEF. Interaction of land use and climate was not included in the model as no significant interaction could be determined. Models including other soil parameters (total carbon, microbial N, N_min_) did not improve the model and were therefore removed. Model goodness of fit statistics were computed using Fisher’s C test. Different models were additionally compared using their AIC values.

## 3 Results

### 3.1 Variability of eco-physiological and microbial activity parameters

**Figure 1:**
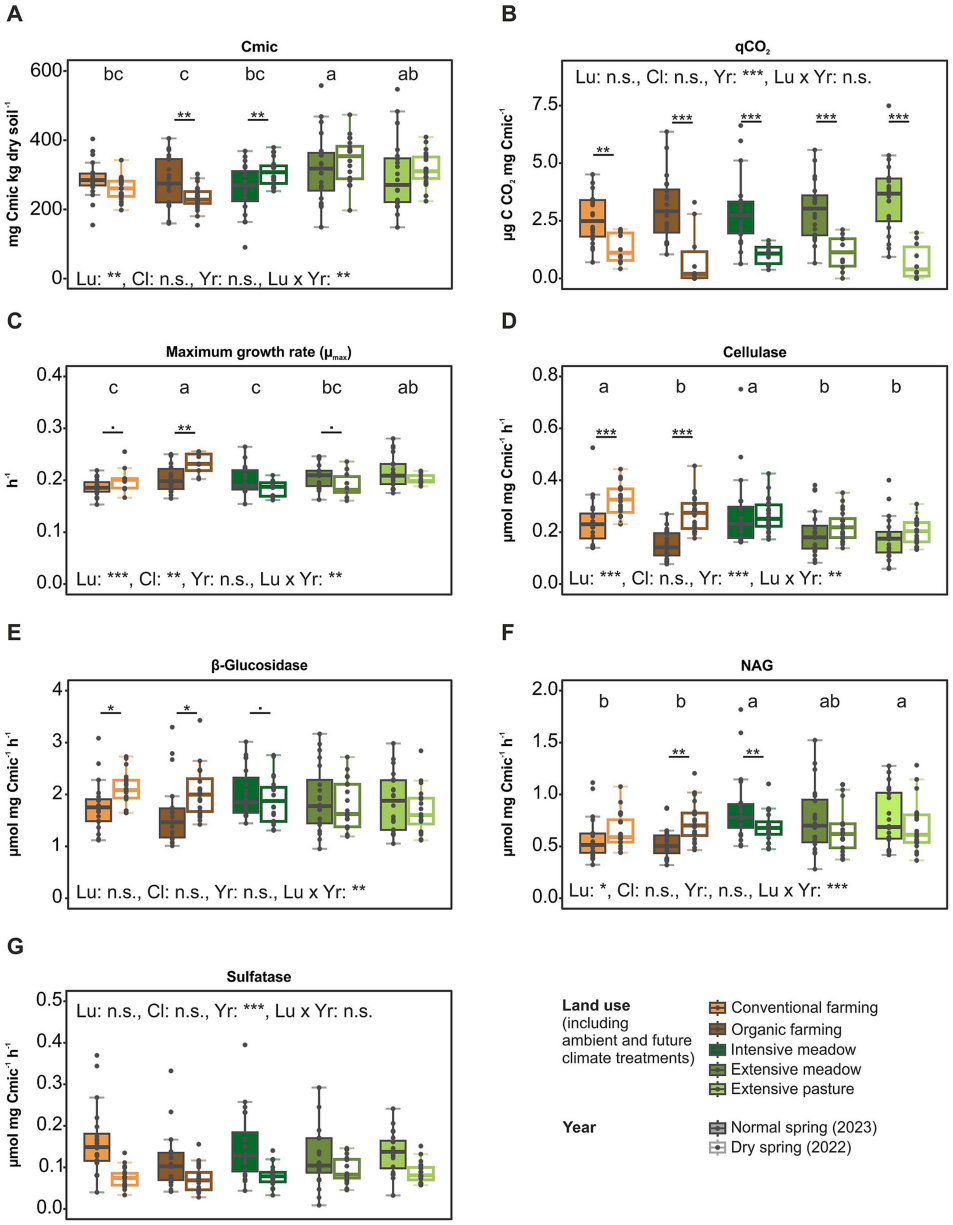
Microbial functional parameters across land-use types in a dry and normal growing season. Microbial biomass C (C_mic_, **A**), metabolic quotient (qCO_2_, **B**), maximum specific growth rate (μ_max_, **C**) and microbial biomass C normalized soil enzymatic activities of cellulase **(D)**, β-glucosidase **(E)**, N-acetylglucosaminidase **(F)** and sulfatase **(G)** are shown. Land-use (Lu), experimental climate treatment (Cl) and year (Yr) and their interacting effects on microbial functional parameters were tested in a three-way ANOVA at significance level *p* = 0.05. Significant differences between land-use types are indicated with letters (a–c), significant pairwise interactions of land-use and year effects are indicated with: ***p < 0.001, **0.001 < *p* < 0.01, *0.01 < *p* < 0.05, •0.05 < *p* < 0.1. Detailed ANOVA results are summarized in Supplementary Table S2.

Among the soil parameters, soil moisture was the most influenced by land use, experimental climate treatment and year (drought). It was lowest in the intensive meadow and highest in organic farming (Supplementary Figures S2, S3; Supplementary Tables S1, S2). In the dry year, soil moisture was 15% lower than in the normal year. There was no effect of experimental climate manipulation in the dry year, while in the normal year, soil moisture was 8% higher under future than under ambient climate. Microbial biomass and microbial quotient (C_mic_/C_org_) were lower in croplands than in grasslands (Figure 1A, Supplementary Tables S1, S2). There was an interacting effect of land use and year, resulting in lower microbial C and C_mic_/C_org_ in croplands than in grasslands in the normal year. The metabolic quotient (qCO_2_) was significantly lower in the dry than the normal year (Figure 1B). Maximum growth rate (μmax) was influenced by both land use and experimental climate treatment, with higher μmax under future than under ambient climate, and the highest μmax in organic farming (Figure 1C; Supplementary Figure S3). Enzymatic activities were unaffected by the experimental climate treatment, but were partly affected by land use with particularly high activities in conventional farming and intensive meadow (Figures 1D–G; Supplementary Figure S1). Most enzyme activities were responsive to the interaction between land use and year (drought). Especially cellulase and β-glucosidase activity exhibited higher activities in the dry than the normal year in croplands, while partly opposite effects were observed in the intensive meadow, with lower NAG and xylosidase activities (Figure 1F; Supplementary Figure S2A).

### 3.2 Microbial community composition

**Figure 2:**
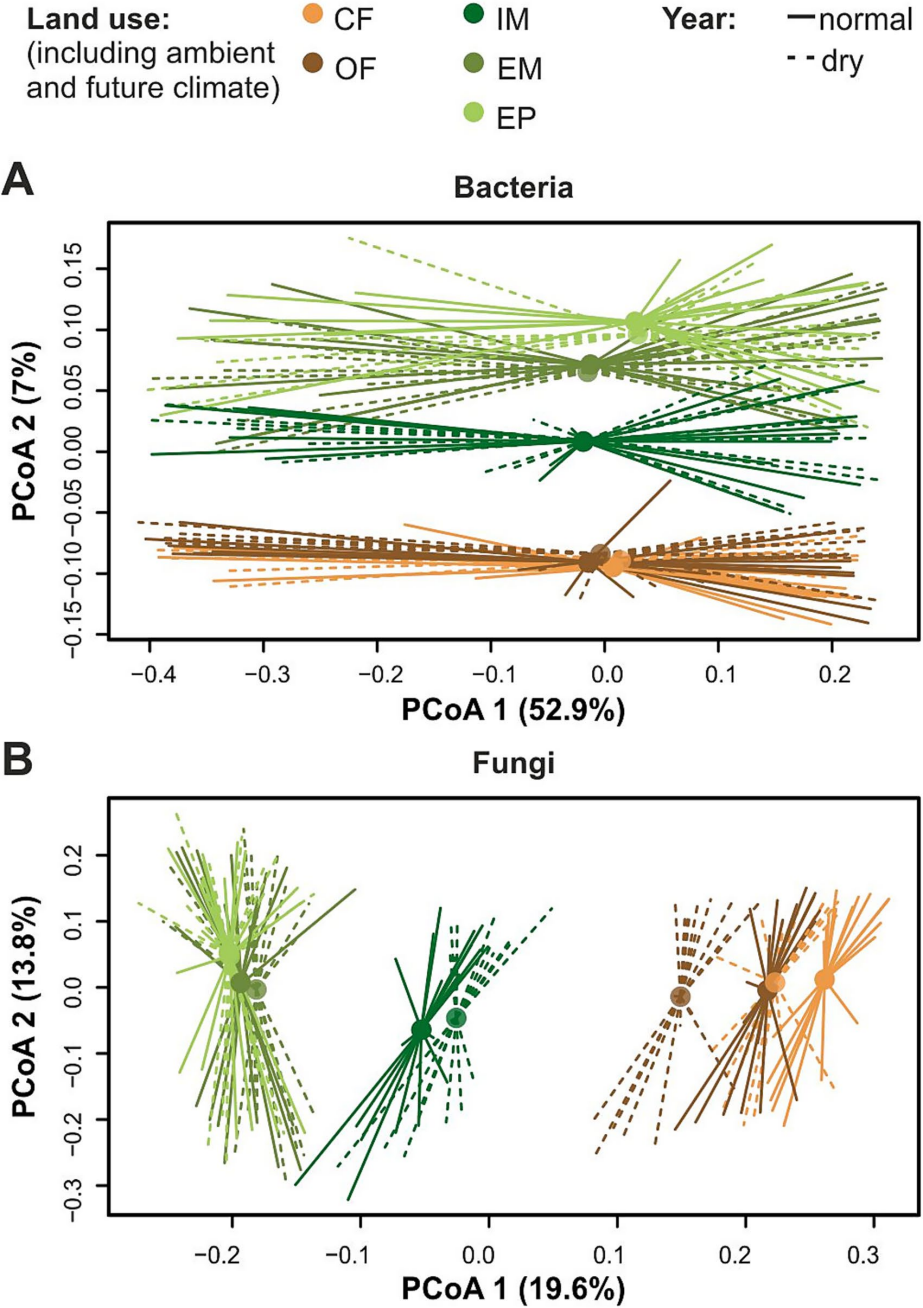
Land-use specific variation between a dry and a normal year in principal coordinate analysis of microbial communities. Principal coordinates were computed from Bray–Curtis dissimilarity index of bacterial **(A)** and fungal **(B)** ASVs after prevalence filtering (1 count in at least 10% of all samples). Samples were grouped by land use type and sampling year. Centroids (shown as circles) are the mean value of PCoA 1 and PCoA 2 of all replicates within the respective group and were plotted with ordispider function in vegan package. Lines and doted-lines indicate the PCoAs of the replicates.

Both bacterial as well as fungal community composition differed between land-use types and between the dry and the normal year (Figure 2; Table 1). Land-use differences were more pronounced in the fungal (*R*^2^: 0.089) than the bacterial community composition (*R*^2^: 0.035). The fungal community composition shifted between the dry and normal year, especially in the two cropland types as well as in the intensive meadow (Figure 2B; Table 1).

**Table 1:** Statistical analysis of fungal and bacterial community composition.

		PERMANOVA bacteria			PERMANOVA fungi		
Factor	Df	*R* ^2^	*F*	Pr(>F)	*R* ^2^	*F*	Pr(>F)
Climate	1	0.015	3.20	0.629	0.015	0.624	0.601
Land use	4	0.035	1.42	**0.046**	0.089	1.95	**0.012**
Year	1	0.004	3.11	**<0.001**	0.011	5.76	**<0.001**

Reported are PERMANOVA results (number of permutations = 9,999), with land use, climate and year as fixed factors, with the bray–curtis dissimilarity the response variable. Statistical significance was determined at *p* = 0.05 and significant results are highlighted in bold.

For bacteria, 62 differentially abundant genera were identified across land-use types (Supplementary Figure S4A; Supplementary Table S3), with the most pronounced differences observed between croplands and grasslands. Few taxa such as *Flavobacterium* were more abundant in grasslands, whereas many taxa (e.g., *Myxococcus*) were more abundant in croplands. There were few differences between conventional and organic farming, though *Devosia* was more abundant in conventional farming. Some taxa differed between the intensive meadow and the extensive grasslands, rendering the intensive meadow more similar to croplands in abundance of some taxa such as *Nitrospira*, *Nitrosospira*, and *Sphingomonas*, −which were found more frequently in the intensive meadow and croplands than in extensive grasslands. In contrast, *Pseudomonas* showed higher abundance in extensive grasslands compared to croplands and *Romboutsia*, *Turibacter* and *Lysinibacillus* were more abundant in the extensive pasture than in the extensive meadow.

For fungi, 66 differentially abundant genera were identified across land-use types (Supplementary Figure S4B; Supplementary Table S3). Many taxa, including *Rhizopus* and *Apodus* were more abundant in croplands than grasslands. In contrast to bacteria, a substantial number of taxa were more abundant in grasslands than in croplands. Differences between conventional and organic farming were more pronounced for fungi than for bacteria. Apart from *Sporomiella* and *Preussia*, which were more abundant in the pasture, extensive meadow and pasture did not differ. Additionally, more fungal taxa differed between the intensive meadow and the extensive grasslands than observed for bacteria. Among those, *Dominika* and *Diversispora* were more abundant in extensive grasslands, whereas *Vishniacozyma* and *Tricellula* were more abundant in intensive meadow than in other land-use types. Experimental climate treatment effects on bacterial and fungal taxa were minimal and not consistent between the dry and normal year (Supplementary Table S4).

### 3.3 Land-use specific differences in microbial taxa between the dry and the normal year

**Figure 3:**
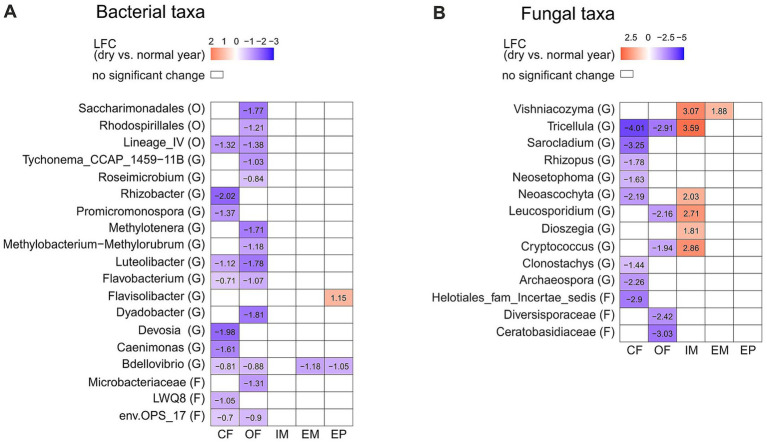
Differentially abundant taxa between the dry (2022) and the normal (2023) year depending on the land-use type. Taxonomic level is indicated in brackets: (G) genus, (F) family and (O) order. Significant changes of bacterial **(A)** and fungal **(B)** taxa are displayed in log2fold change (LFC). Significant differences were determined with Dunnett’s type of test *p* = 0.05. Abbreviations for land-use types are: CF, Conventional farming; OF, Organic farming; IM, Intensive meadow; EM, Extensive meadow; EP, Extensive pasture.

Bacterial and fungal taxa showed a land-use specific difference in abundance between the dry and the normal year. The majority of inter-annual differences was identified in the croplands, all of which showed a lower abundance in the dry year than in the normal year (Figure 3A). The taxa *Deviosa*, and *Clonostachys* were reduced in conventional farming, while *Roseimicrobium* was reduced in organic farming, and *Tricellula*, *Flavobacterium* and *Luteolibacter* were reduced in both croplands. No year related differences in bacterial taxa were found in the intensive meadow. In contrast to croplands, in the intensive meadow several fungal genera, such as *Vishniacozyma*, *Tricellula* and *Neoascochyta* were more abundant in the dry year than the normal year (Figure 3B). Some of these microbial taxa, were correlated with soil moisture. *Deviosa*, *Clonostachys*, *Neoascychota* and *Vishniacozyma* showed a weak negative, and *Flavobacterium* and *Roseimicrobium* showed a weak positive correlation (Supplementary Table S5).

### 3.4 Trophic modes of fungi

**Figure 4:**
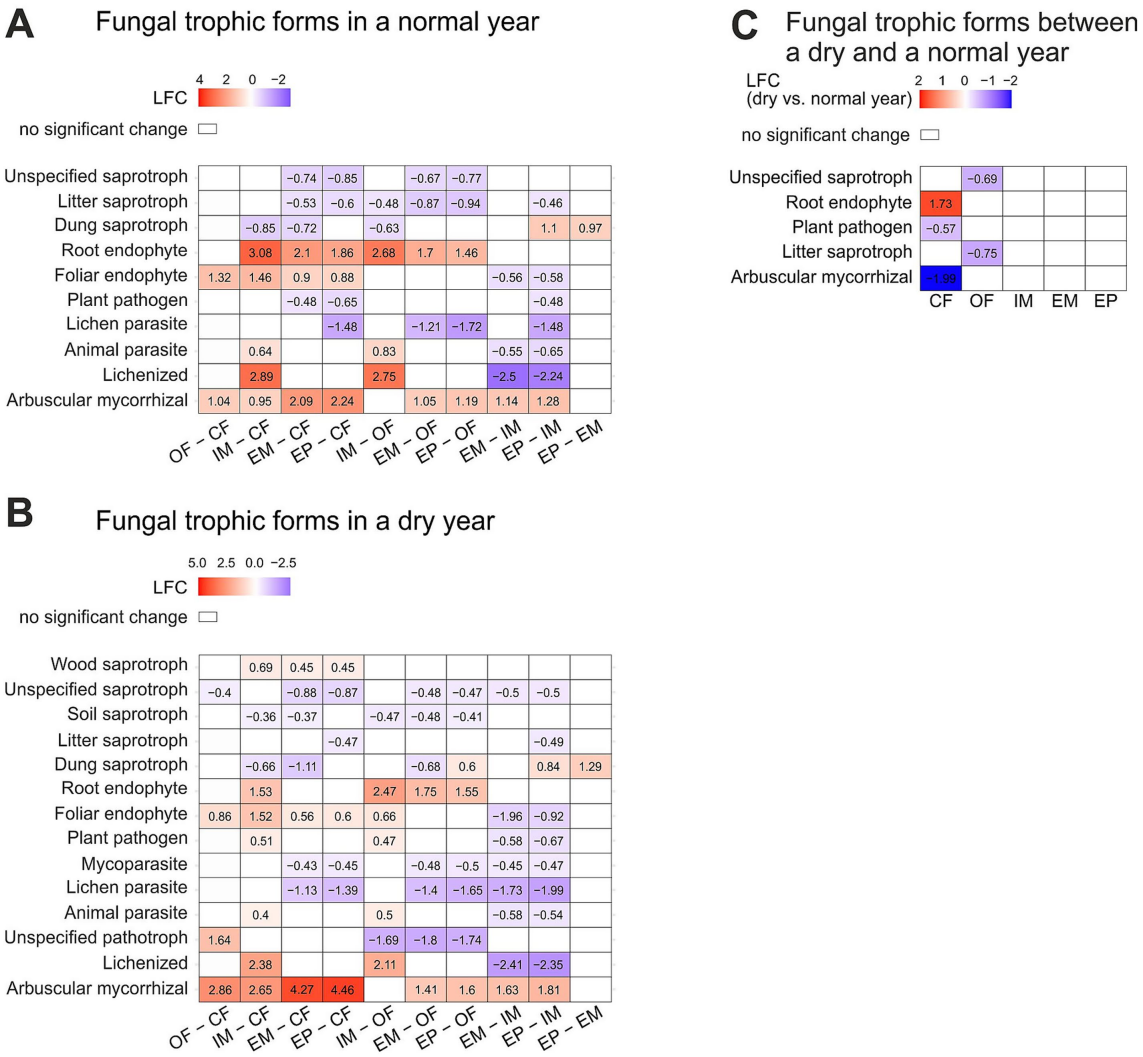
Differentially abundant fungal trophic forms between land-use types a normal **(A)** and a dry **(B)** year and land-use specific changes between a dry and normal year **(C)**. Significant changes are displayed in log2fold change (LFC). Significant differences were determined with Dunnett’s type of test at *p* = 0.05. For detailed summary see Supplementary Table S6. Abbreviations for land-use types are: CF, Conventional farming; OF, Organic farming; IM, Intensive meadow; EM, Extensive meadow; EP, Extensive pasture.

Trophic modes of fungi differed between land-use types especially in the dry year (Figure 4). While between extensive meadow and extensive pasture, the sole difference was in dung saprotrophic fungi with higher abundance in the pasture, there were many pairwise differences between all other land-use types. In the normal year, only a few trophic modes of fungi differed between land-use types, including saprotrophic fungi which were more abundant in croplands than in extensive grasslands (Figure 4A). In the dry year, more differences between land-use types were observed: soil and unspecified saprotrophs were more abundant in croplands than in extensive grasslands (Figure 4B). Parasitic and pathogenic fungi were less abundant in extensive grasslands over the dry and normal year. In contrast, plant pathogens and animal parasites, were most abundant in the intensive meadow particularly in the dry year, with higher abundance even than in conventional farming. In contrast, arbuscular mycorrhizal fungi were more abundant in extensively managed systems. Land-use specific changes between the dry and the normal year were found in croplands, but not in grasslands. In conventional farming, plant pathogenic fungi were less abundant in the dry than the normal year. For organic farming, litter saprotrophic fungi were less abundant in the dry year (Figure 4C).

### 3.5 Co-occurrence networks

**Figure 5:**
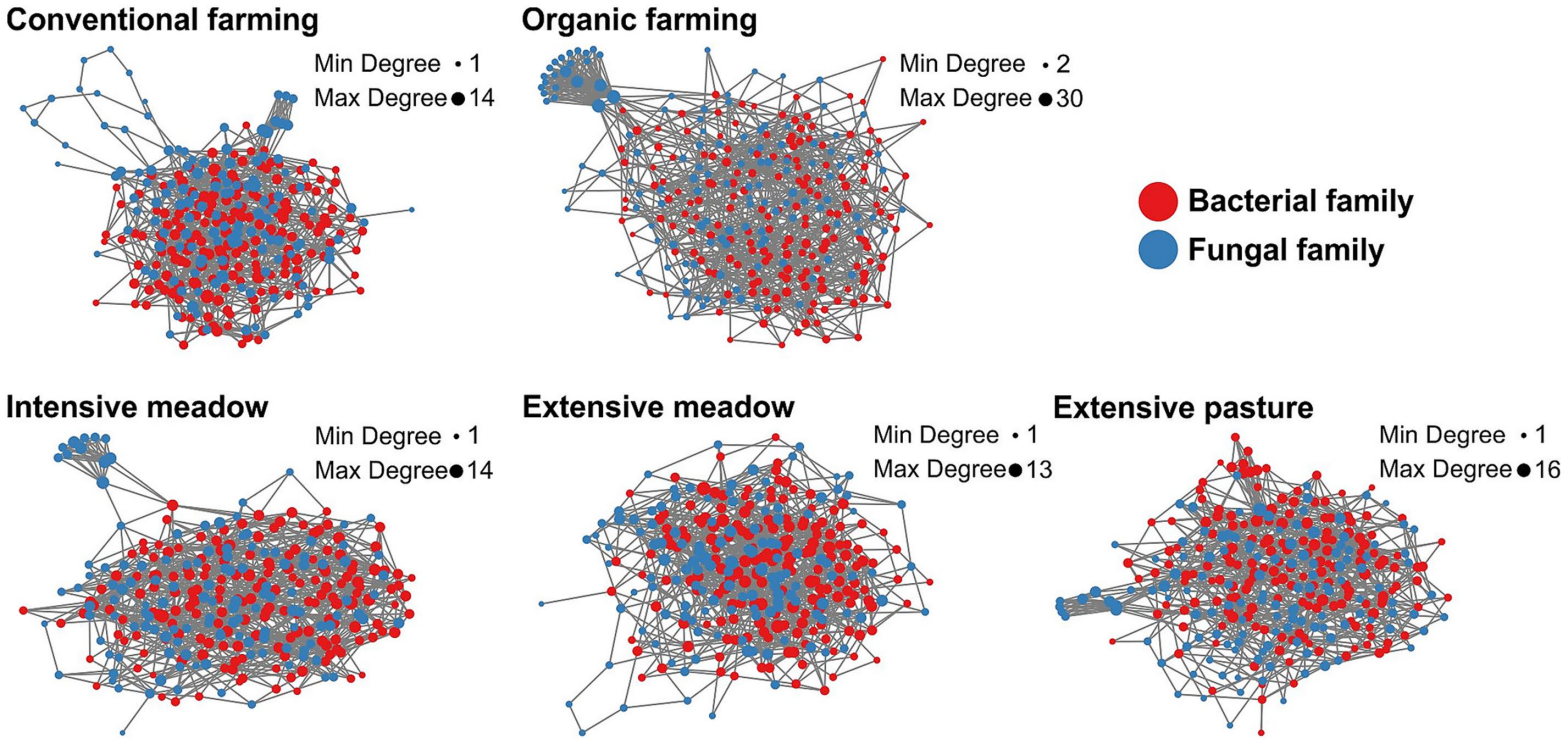
Co-occurrence networks of fungal and bacterial families in different land-use types in a normal year. Each node represents one family, the size of the node refers to the degree, minimum and maximum degree (Min Degree and Max Degree) within the given network are shown. More network properties are summarized in Tables 2, 3 and Supplementary Tables S7, S8. Co-occurrence networks were constructed for the dry year as well (Supplementary Figure S5).

Species co-occurrence networks of fungal and bacterial taxa exhibited structural differences between land-use types with lowest modularity in intensive and extensive meadows and highest average path length in conventional farming (Table 3). While certain network features within a given land-use type remained similar, the percentage of positive edges was lower in croplands, and intensive meadows in the dry compared to the normal year. Hub taxa differed between all land-use types (Table 2; Supplementary Table S6). In organic farming, fungi formed a very prominent cluster within the network (Figure 5; Supplementary Figure S5). Consequently, the number of edges between fungal taxa was higher in organic farming than in other land-use types especially in the normal year, while the share of cross-domain edges between prokaryotic and fungal taxa was low (Table 3). Conventional farming exhibited the lowest average degree and the fewest edges of all land-use types (Table 3). A fungal cluster was present in most land-use types, except in extensive meadows, where no prominent clusters of fungi or bacteria were identified in either year. Despite differences in taxa composition, the dominant functional groups in the cluster were similar across land-use types, with soil, litter and wood saprotrophic forms being most abundant (Supplementary Table S7). Similarly, the fungal taxa with the highest degree across the entire network were mostly soil, litter or wood saprotrophs as well (Supplementary Table S8).

**Table 2:** Land-use specific comparison of co-occurrence network properties between a dry and a normal year.

	CF dry vs. normal year		OF dry vs. normal year		IM dry vs. normal year		EM dry vs. normal year		EP dry vs. normal year	
Network property	Jacc	*P* value	Jacc	*P* value	Jacc	*P* value	Jacc	*P* value	Jacc	*P* value
Degree	0.13	**<0.001**	0.18	**<0.001**	0.13	**<0.001**	0.16	**<0.001**	0.18	**0.001**
Betweenness centrality	0.23	**0.006**	0.18	**<0.001**	0.11	0	0.23	**0.006**	0.19	**<0.001**
Closeness centrality	0.25	**0.023**	0.25	**0.023**	0.16	**<0.001**	0.27	0.071	0.29	0.173
Eigenvector centrality	0.10	0	0.23	**0.006**	0.17	**<0.001**	0.29	0.173	0.21	**0.001**
Hub taxa	0	**0.012**	0.09	0.075	0.00	**0.003**	0.08	**0.039**	0	**<0.001**

Jaccard similarity index (Jacc, Jaccard index of 1 indicates 100% similarity of data sets) was calculated to determine the difference in network properties in land uses [conventional farming (CF), organic farming (OF), intensive meadow (IM), extensive meadow (EM) and extensive pasture (EP)] between a dry and normal year. Statistical significance was determined at *p* = 0.05 using the NetCoMi package, and significant results are highlighted in bold.

**Table 3:** Microbial co-occurrence network properties in a dry and a normal year depending on land-use type.

	Normal year					Dry year				
	CF	OF	IM	EM	EP	CF	OF	IM	EM	EP
Total nodes	303	303	303	303	303	303	303	303	303	303
Total edges	872	956	951	911	908	894	922	951	931	923
% total negative edges	40.0	37.2	42.4	42.9	42.6	42.8	41.5	47.1	43.7	43.2
% edges between bacteria and fungi	40.1	35.6	40.9	40.1	39.8	34.2	37.3	40.7	41.2	41.7
% edges within bacteria	38.1	38.7	38.3	39.8	38.7	42.3	39.3	38.4	38.3	39.4
% edges within fungi	21.8	25.7	20.8	20.1	21.6	23.5	23.4	20.9	20.4	18.9
Modularity	0.46	0.47	0.47	0.46	0.5	0.5	0.49	0.46	0.46	0.48
Average degree	5.76	6.31	6.28	6.01	5.99	5.9	6.09	6.28	6.14	6.09
Average path length	2.58	2.48	2.50	2.49	2.55	2.59	2.53	2.44	2.46	2.52

Listed are the network properties in five land-use types conventional farming (CF), organic farming (OF), intensive meadow (IM), extensive meadow (EM) and extensive pasture (EP). All parameters were computed with the NetCoMi package, edge percentages were derived from association matrices.

### 3.6 Structural equation modeling

**Figure 6:**
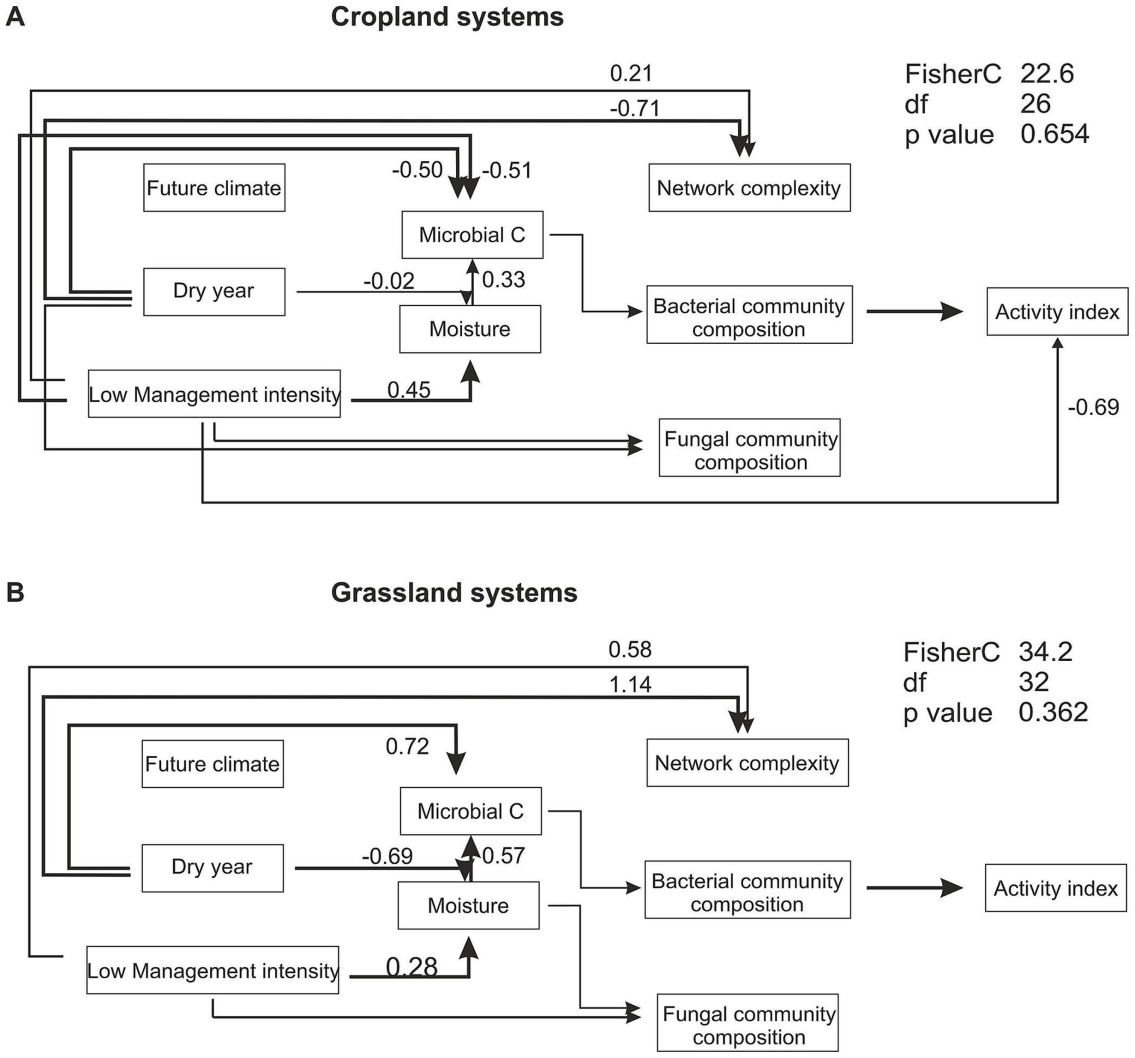
Structural equation modeling of land use, experimental climate treatment and year (drought) effects on soil and microbial parameters. Separate models were created for croplands **(A)** comprising conventional and organic farming and grasslands **(B)** comprising of intensive and extensive meadow. Within the respective model organic farming and extensive meadow were the low management intensity types. Significant paths at *p* = 0.05 with arrows indicating directionality and the respective path estimates are shown. The activity is a summary score of all measured enzymatic activities. Detailed information on calculation of indices can be found in the method section. Estimates for all paths, as well as land-use type specific path estimates within the SEM are summarized in Supplementary Table S9. The overall model fit was assessed with the Fisher’s C test, with *p* > 0.05 indicating a good model fit.

Structural equation modeling revealed significant relationships between land-use, and year (drought) with environmental variables, community diversity and functions (Figure 6; Supplementary Figure S6; Supplementary Table S9). SEMs were constructed for croplands and grasslands separately, revealing differential relationships between the variables. In croplands, low management intensity (organic farming), positively affected soil moisture, but negatively affected microbial C content. The dry year had a minimal negative influence on soil moisture, but a great negative influence on microbial C content. In contrast to bacterial community composition, the fungal community composition was directly influenced by dry year and low management intensity. Microbial co-occurrence complexity was influenced by dry year negatively and by low management intensity positively. Interestingly, microbial activity index was only influenced by bacterial community composition, and low management intensity. In the grassland SEM, similarly to the cropland SEM low management intensity (extensive meadow) had a positive relationship with soil moisture. In contrast to croplands, the dry year had a strongly negative relationship with soil moisture, but a strong positive effect on microbial carbon content. Microbial community composition in grasslands showed similar relationships with other variables as in croplands, except for fungal community composition, which was not affected by the dry year. The microbial activity index was influenced by bacterial community composition only. Additionally, the SEM was constructed including all land-use types. While many of the observed relationships remained similar, the network complexity variable could not be integrated into a meaningful model. Microbial C was influenced by land-use type and did positively influence the activity index, which was not the case for neither the cropland nor the grassland model. In all constructed SEMs, the experimental climate treatment did not affect any variable. Other soil parameters, as well as an abundance index of fungal trophic groups did not improve the model or did not meet the goodness of fit criteria and were therefore not used in the final model construction.

## 4 Discussion

### 4.1 Extreme event effects outweigh long-term future climate effects

The effects of the experimental future climate treatment on microbial parameters, such as biomass, enzymatic activities, metabolic quotient, and community composition were much less pronounced than the effects of the drought in 2022. The future climate treatment at the study site simulates a slight warming with a mean temperature increase of 0.55°C and an increase in spring precipitation of 10%. Unlike many climate manipulation experiments that impose severe drought or temperature regimes (Korell et al., 2020), the mild GCEF treatment reflects realistic climate projections, consistent with model-based simulations of the future climate for the region (Schädler et al., 2019). Despite the conservative changes, the future climate has been shown to influence plant and soil parameters, as well as invertebrate and microbial communities and their biological activities (Yin et al., 2020; Kostin et al., 2021; Korell et al., 2024). In this study, the maximum specific growth rate of microorganisms was the only parameter that was affected by the experimental climate treatment in both years, regardless of spring conditions. These results suggest a higher potential for microbial growth under the predicted future climate as it has been previously observed by Rousk et al. (2013). Higher growth potential may come with the cost of lower stress tolerance and C resource acquisition capacity of microbes, potentially lowering C use efficiency and altering soil C cycling (Malik et al., 2020; Osburn et al., 2024). The year-to-year comparison in this study included one dry (2022) and one normal (2023) spring season. In the dry year, the precipitation amount was only 30% of the precipitation in the normal year, and only 38% of the long-term mean precipitation (1997–2021) at the site, highlighting its nature as a climatic extreme. This strong inter-annual difference affected the magnitude of the experimental climate effects, which were less pronounced in the dry than the normal year. Specifically, soil moisture was higher under future climate in the normal year, while no such effect was observed in the dry year, confirming our first hypothesis. Under the extremely dry conditions in 2022, even the favorable climate manipulation, e.g., a wetter and warmer spring, does not compensate for the detrimental effects of water limitation. In such years, critical soil moisture thresholds required to sustain microbial activity are not reached, thereby constraining any potential climate-induced stimulation. Our findings suggest that climatic extremes play a more substantial role in shaping microbial community composition and functions than the simulated mean climate change. This aligns with similar observations reported for litter microbial communities (Matulich et al., 2015). The finding is reflected in the structural equation model, which shows that the extremely dry spring strongly affected soil microbial parameters in croplands as well as grasslands. The year-specific differences in the precipitation amount likely reflect broader climate change trends, i.e., increasing weather extremes (Kahraman et al., 2021; Goffin et al., 2024), more frequent droughts (Spinoni et al., 2018; Böhnisch et al., 2021) and higher precipitation variability (Pendergrass et al., 2017). These results highlight the need to consider extreme events in addition to mean changes when investigating the microbial response to global climate change.

### 4.2 Grasslands showed lower sensitivity to extreme drought

Grasslands and croplands represent contrasting ecosystems with differences in plant communities and management practices, which both influence soil microbial communities. This contrast was reflected by strong differences in microbial biomass, community composition, and functional potential. We found typical indicator taxa in the different land-use types. *Myxococcus* species that have been described as cropland indicator species (Gschwend et al., 2022) were more abundant in croplands, while *Romboutsia*, *Turibacter*, and *Sporomiella* were selectively increased in the extensive pasture, which is consistent with other grazing related studies (Wood and Wilmshurst, 2012; Sánchez-Marañón et al., 2024).

Generally, the dry spring caused lower soil moisture levels and lower metabolic quotients across all land-use types indicating lower availability of labile C under these extreme conditions. Microorganisms may adapt to moisture shifts through changes in community composition and functional traits, altering microbial processes such as nitrogen and carbon cycling (Bimüller et al., 2014; Fuchslueger et al., 2016; Zhou et al., 2020). Drought stress can lead to the selection of drought-resistant microbial taxa, e.g., certain bacteria or fungi that can form dormant spores (Manzoni et al., 2014), while other taxa are favored under normal conditions. Grasslands, dominated by perennials, and croplands, characterized by annual crops differ in the amount of root exudation, and exudate quality under drought depending on plant growth strategies (Ouédraogo et al., 2013; Williams and de Vries, 2020). These plant-driven differences in litter input and root exudates could modify microbial responses to dry or normal conditions (Karlowsky et al., 2018). Croplands often experience more dynamic plant-microbe interactions caused by fluctuations in nutrient inputs due to the annual crop life cycle, fertilizer input and tillage, potentially leading to faster microbial growth, adaptation and turnover of microbial populations (Degrune et al., 2017; Wang et al., 2021; Wang et al., 2022). Consequently, changes in the abundance of individual bacterial and fungal taxa between the extreme drought and the normal year occurred primarily in croplands confirming our second hypothesis that grasslands are more resilient to extremes than croplands. Interestingly, the fungal community composition was much more sensitive to the extreme drought than the bacterial community composition, with shifts observed in croplands but also in the intensive meadow. The observed sensitivity of the intensive meadow suggests that intensive management can diminish the buffering capacity of grassland systems, making them respond to climate extremes in a similar way as croplands. While the crop rotation may partly explain the pronounced changes between the years in croplands due to the cultivation of different crop species, similar changes in the intensive meadow that is characterized by perennial forage grasses suggest that the primary driver was the extreme drought with the different precipitation and soil moisture conditions. Fungal community functions also differed in croplands between the dry and normal year, indicating that fungal communities play a key role in mediating soil responses to climate extremes. To our surprise we did not find a significant link between fungal community composition and enzymatic activity in our SEMs. We assume that this is partly due to how fungal communities were represented in the model. We used the first PCoA axis as a proxy which captured only 20% of the variation likely not fully reflecting relevant aspects of fungal diversity and highlighting a common issue in microbial ecology to link structure to biological function. Future studies should explore alternative ways to assess community data to more adequately capture the relevance of fungi in SEMs. Despite the lack of a significant structure–function link in the SEM, patterns in the relative abundance of specific fungal taxa suggest ecologically relevant shifts in functional composition. High abundance of fungal decomposers like *Rhizopus* species, with a wide array of extracellular enzymes (Lennartsson et al., 2014) and *Apodus* species, associated with N starved plant rhizospheres (Pagé et al., 2019) in croplands, reflect a decomposer dominated community in croplands. Such communities may be more susceptible to changes in environmental conditions and lower substrate availability in drought as we found lower abundance of saprotrophic fungi in organic farming in the dry year. Community shifts may be linked to differences in microbial biomass and enzyme activities. Interestingly, in croplands, C-cycling enzyme activities were higher in the dry spring than in the normal spring, whereas they remained unchanged in grasslands. Two potential mechanisms could explain this pattern. First, dry conditions across the whole year 2022 at the study site (38% less than average) may have reduced plant productivity and litter quality (Liu et al., 2023), leading to prolonged substrate scarcity, particularly in the grasslands in the normal year. This limitation could have constrained microbial activity, as we observed no change in grassland microbial biomass between the dry and the subsequent normal spring season. Second, higher soil moisture in the normal year may have caused higher nutrient availability. Mineral N availability is known to increase with higher soil moisture levels (Li et al., 2009; Månsson et al., 2014), which is consistent with our findings. This could explain the lower enzyme activity observed in croplands in the normal year, as microbial communities may have relied on directly available nutrients rather than on investing in enzyme production. In contrast, the competition for nutrients with plants may have increased especially in grasslands, forcing microbes to invest in enzyme production. Further, higher soil moisture may have promoted the abundance of fauna grazing on microbes explaining microbial biomass stability over dry and normal spring conditions. Overall, grasslands, with some exceptions in the intensive meadow, were less responsive to climatic extremes, largely confirming our expectation that grasslands will be less sensitive to climate extremes than croplands.

### 4.3 Land use and fertilization regime shape microbial community composition

Differences between management intensity (e.g., conventional vs. organic farming and intensive meadow vs. extensive meadow) were supported by the structural equation models, indicating higher soil moisture under low-intensity management, taxonomic shifts and altered network structures. While both, drought and management intensity, affected soil and microbial parameters, we generally did not find stronger drought effects on intensively managed systems. However, stress tolerant bacterial taxa such as *Sphingomonas* and *Deviosa*, known for their ability to metabolize herbicides (Asaf et al., 2020; Talwar et al., 2020; Ruuskanen et al., 2023), were more abundant in both intensive systems, i.e., in conventional farming and intensive meadow, likely using herbicide residues as substrates. Moreover, the pathogenic potential increased under intensive management of conventional farming and intensive meadow indicating a shift away from mutualistic communities. Pesticide use and intensive management can facilitate the establishment of potentially harmful microbial taxa (Lekberg et al., 2021), thereby increasing the risk of losing ecosystem functions, such as productivity and plant diversity. Although we did not find consistent drought-induced shifts of pathogenic taxa, this issue may become more relevant in the future, as some plant pathogens are more resilient to global warming (Rissi et al., 2024) and the presence of new fungal pathogens has been documented for conventional farming under future climate in this experiment (Fareed Mohamed Wahdan et al., 2020). In contrast, extensive grasslands supported beneficial taxa such as *Pseudomonas*, a prominent plant growth promoting genus, as well as arbuscular mycorrhizal fungi (*Dominika*, *Diversispora*). This suggests that extensive management induces greater reliance on internal nutrient cycling and more stable plant–soil interactions, potentially contributing to higher resistance under experimental climate change and the extreme drought in extensive grasslands. Taxa related to nitrification, such as *Nitrospira* and *Nitrosospira* were more abundant in croplands and the intensive meadow, indicating higher N availability for nitrification or a community specialized for N cycling (Schramm et al., 1998; Koch et al., 2015). Furthermore, our data indicated stability of the N cycling communities as no differences between the dry and normal spring season were observed.

Interestingly, individual bacterial and fungal taxa were affected by the dry year in both cropland types, while in grasslands, only under intensive management several fungal taxa showed higher abundance in the dry year. Three of those taxa *Vishniacozyma*, *Tricellula* and *Neoascochyta* had been positively correlated with C content in wheat straw (Shamshitov et al., 2025), pointing toward a drought-induced difference in the nutrient content of plant biomass in intensive meadows. Other differentially abundant taxa were assigned to *Flavobacterium*, which has been associated with high drought tolerance (Gontia-Mishra et al., 2016), *Vishniacozyma* that can persist under harsh environmental conditions (Botha et al., 2024), as well as *Clonostachys* and *Bdellovibrio*, which have been associated with parasitic or predatory lifestyles and were described as potential biocontrol agents (Bratanis et al., 2020; Sun et al., 2020). Although these taxa could provide important functions, no consistent functional trends or correlation with soil moisture were observed. These results emphasize that management intensity, in addition to land-use type, influences microbial community susceptibility to climate change and extreme events, with intensively managed systems, croplands and intensive meadows, exhibiting more compositional shifts.

We expected that land-use intensification would reduce microbial network complexity. Our results presented a more nuanced picture. Extensive grasslands appeared more stable in their co-occurrence networks, with a more consistent number of positive associations under both, dry and normal spring conditions. Yet, overall network complexity as indicated by edge number was highest in organic farming and intensive meadows. However, modularity and average path length were lowest in extensive meadows, suggesting that extensive grasslands exhibit more cohesive and resilient networks, whereas intensively managed systems show more variable and compartmentalized networks. These findings highlight that network complexity is a multidimensional concept. Positive co-occurrences—often interpreted as cooperative or synergistic interactions (Xun et al., 2017; Zhang et al., 2018)—were more frequent in croplands and the intensive meadow in the normal year than the dry year. This shift may imply that intensively managed systems are more sensitive to climate extremes, as more positive associations can also reflect destabilization under stress (Hernandez et al., 2021). Their increase in the normal year points to potential legacy effects of the preceding drought.

Across all land-use types, each network was shaped by distinct keystone taxa, which also differed between the dry and normal year. Keystone or hub taxa are not necessarily the most abundant taxa, nor are they necessarily the most beneficial species themselves (Shade et al., 2014), but play important roles in the assembly of plant beneficial communities (Toju et al., 2018) or serve as indicators for broader shifts in microbial community composition (Herren and McMahon, 2018). Many of the connected key taxa shared potential traits (e.g., saprotrophic fungi), suggesting that microbial communities maintained core functions despite differing environmental conditions between the dry and normal year. In contrast to other reports, not only prokaryote-to-prokaryote edges (Mo et al., 2024), but also prokaryote-to-fungi edges dominated the networks, highlighting the importance of both fungal and bacterial communities. In our case, greater edge density in organic farming may not indicate functional robustness, but could reflect the formation of isolated prokaryote- and fungus-specific niches (Röttjers and Faust, 2018), as suggested by the lower proportion of cross-domain interactions. Such compartmentalization could limit metabolic complementarity and thereby reduce the overall functional potential. This interpretation aligns with consistently low enzymatic activity potentials found in organic farming in this and in previous work at the site (Breitkreuz et al., 2021; Philipp et al., 2025). Thus, in depth microbial network analysis including fungal as well as bacterial taxa is a potential tool to reveal an effect of domain-specific niches on the microbial community functioning in light of changing climate and management regimes (de Menezes et al., 2015; de Menezes et al., 2017).

## 5 Conclusion

Our findings demonstrated that climatic extremes have a much stronger effect on soil microbial communities than mean climate change does. This highlights the need to integrate both aspects into climate change studies in order to reveal the impact of a future climate on agroecosystems. Extreme drought, primarily altered soil moisture, which influenced microbial biomass and activity, with differential responses between land-use types and management intensity. Extensively managed grasslands showed greater resistance, while intensive meadows partly responded similarly to croplands, suggesting that intensive management diminishes grassland resistance to drought events. Although several community and network shifts occurred under extreme drought, many of these changes could be neither linked directly to soil moisture nor to specific functions of the microorganisms. This indicates that microbial communities may adapt structurally, while maintaining functional stability. However, further research is required to understand global change effects on soil microbial communities in more detail. Given the limited two-year study period we can only carefully infer conclusions about resistance and resilience of microbial communities - as resistance and resilience can only adequately be captured over longer time scales. Specifically, considering the projected increase in frequency of extreme events such as droughts or heat waves, future work should explore how repeated stressors will impact microbial community stability and recovery in terms of structure and function. Furthermore, our tools to determine microbial functions were limited to measure enzymatic activity and inference of functional groups with bioinformatic tools. Future studies should focus on analyzing expression of genes related to stress responses and to nutrient cycling. Such molecular methods would capture functional changes in more detail and could help to bridge the gap to link structural and functional changes of soil microbial communities in response to global change.

## Data Availability

The datasets presented in this study can be found in online repositories. The names of the repository/repositories and accession number(s) can be found in the article/Supplementary material.
