# Supplementary material for: Soil microbial communities are more disrupted by extreme drought than by gradual climate shifts under different land-use intensities
Source: Front Microbiol. 2025 Aug 7;16:1649443. doi: 10.3389/fmicb.2025.1649443 (PMC12367804; doi:10.3389/fmicb.2025.1649443)
Supplement: Supplementary file 2 [file Data_Sheet_1.docx]

**Supplementary material**

**Soil microbial communities are more disrupted by extreme drought than by gradual climate shifts under different land-use intensities**

Data availability:

Raw sequencing reads can be found in the NCBI Sequencing Read Archive (SRA) under the accession numbers: PRJNA1277715 and PRJNA1234075.


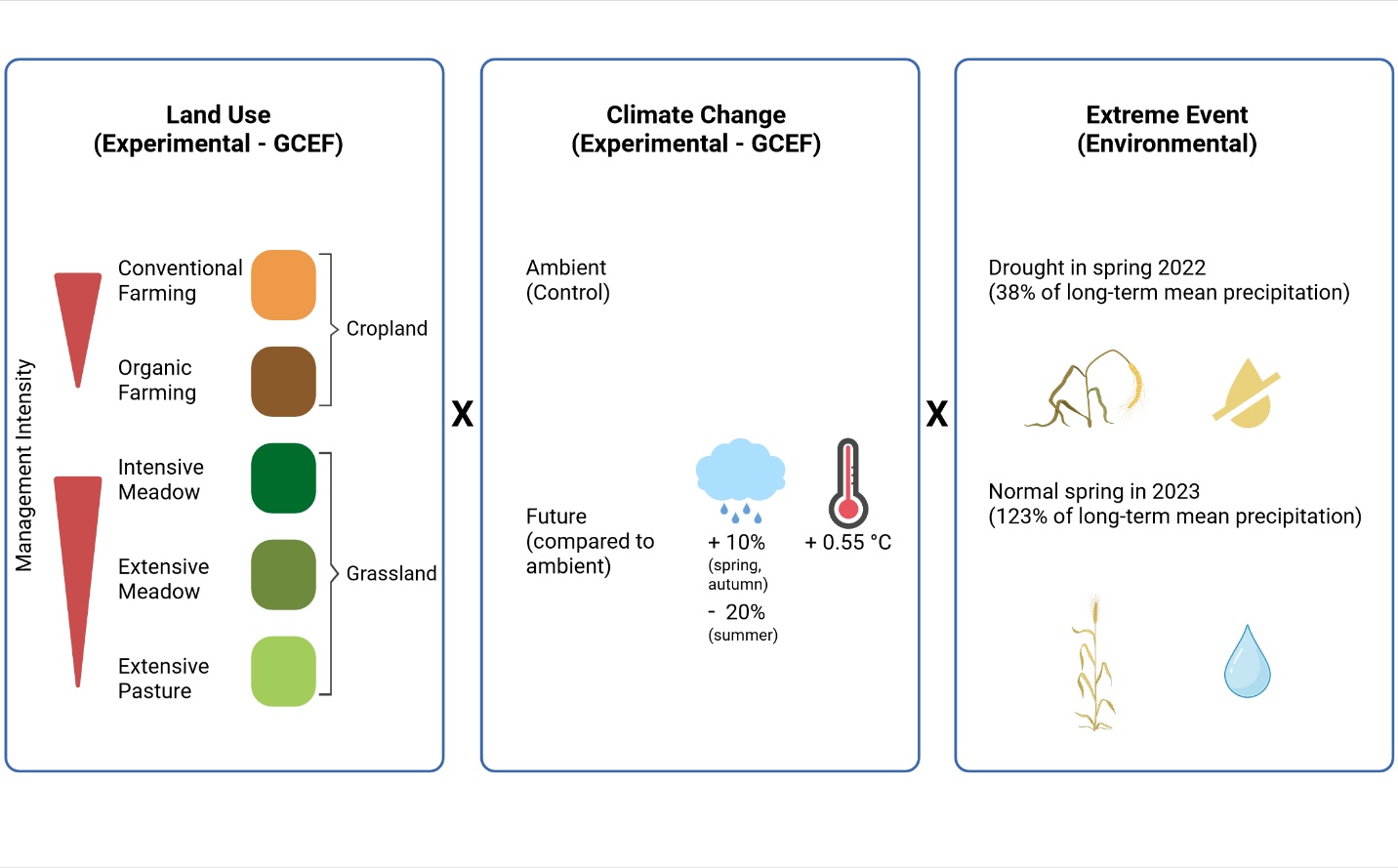
Supplementary Figure S1: Experimental design of the study. Three global change factors and their interactions were investigated. The specificities of the two experimental factors land use and experimental climate change in the Global Change Experimental Facility (GCEF) as well as the environmental factor (the extreme event) are summarized. This figure was created in BioRender. Philipp, L. (2025) https://BioRender.com/4hvtv05.


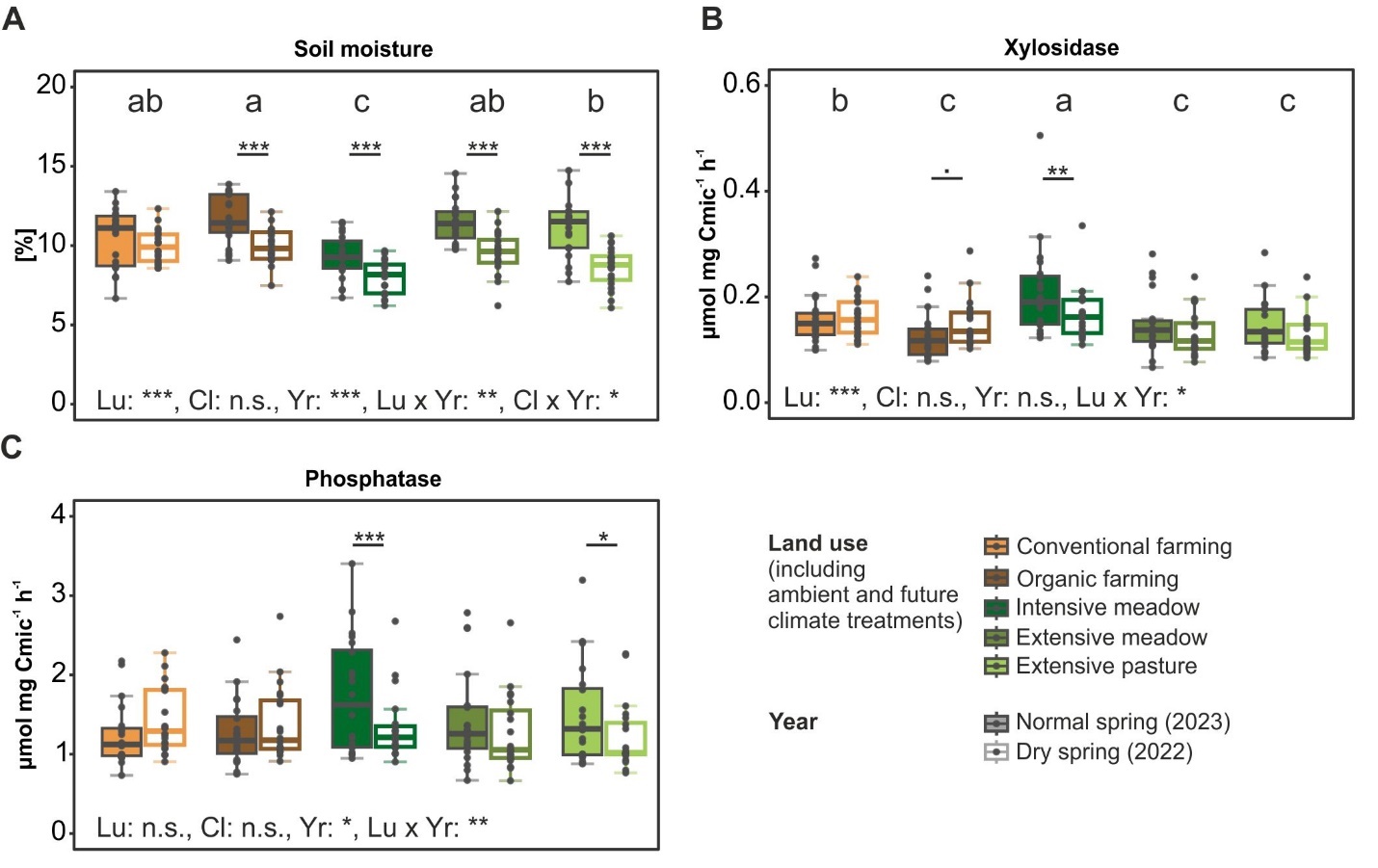


Supplementary Figure S2: Soil moisture and microbial functional parameters across land-use types in a dry and wet growing season. Soil moisture (A) and microbial biomass C normalized enzymatic activities of xylosidase (B) and acid phosphatase (C) are shown. Land-use (Lu), climate (Cl), year (Yr) and their interacting effects on microbial functional parameters were tested in a three-way ANOVA at significance level p = 0.05. Significant differences between land-use types are indicated with letters (a-c), significant pairwise interaction of land-use and year effects are indicated with: *** p<0.001, **, 0.001 <p<0.01, * 0.01 < p < 0.05, . 0.05<p<0.1. Detailed ANOVA results are summarized in Supplementary Table S2.


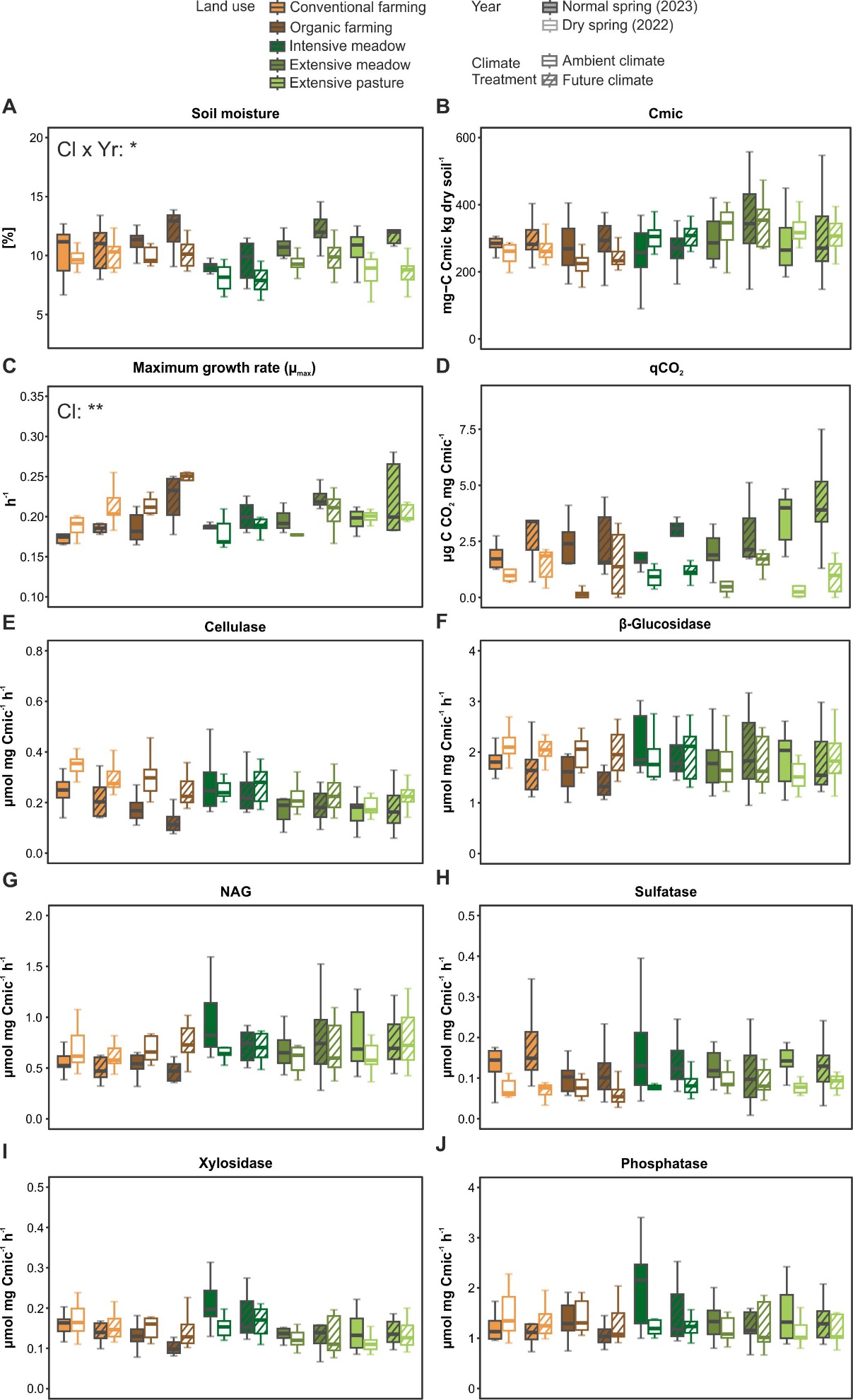


Supplementary Figure S3: Soil moisture and microbial functional parameters across land-use types and experimental climate treatment in a dry and a normal growing season. Soil moisture (A), microbial biomass (B), maximum specific growth rate (C), metabolic quotient (D) and microbial biomass C normalized enzymatic activities (E-J) are shown. Land-use (Lu), experimental climate treatment (Cl), year (Yr) and their interacting effects on microbial functional parameters were tested in a three-way ANOVA at significance level p = 0.05. For clarity, only significant ANOVA results for experimental climate treatment are reported in the figure, as all other significant results are shown in Figure 1 and Supplementary Figure S2. Detailed ANOVA results are summarized in Supplementary Table S2.


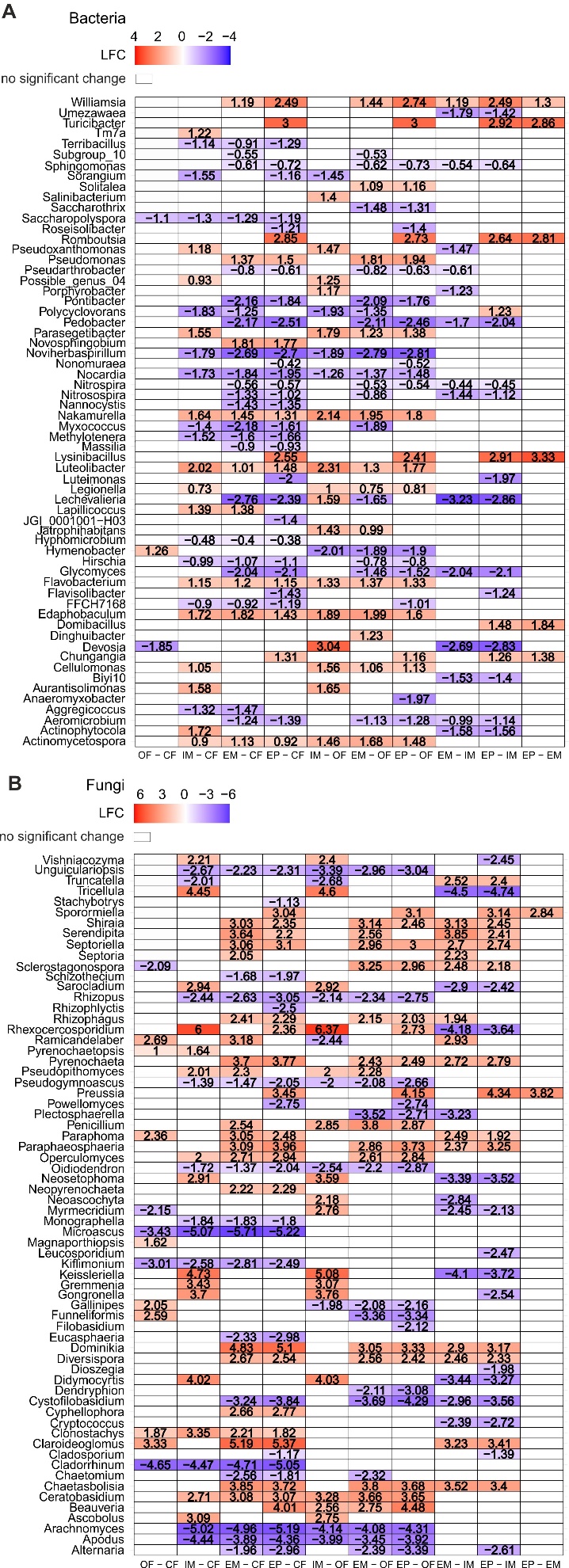


Supplementary Figure S4: Differentially abundant microbial taxa between pairwise land-use type comparison based on ANCOMBC2 analysis. Significant differences of bacterial (A) and fungal (B) taxa are displayed as log2fold change (LFC). Significant differences were determined with Dunnett’s type of test at p = 0.05. Detailed test results and standard error values are summarized in Supplementary Table S3.


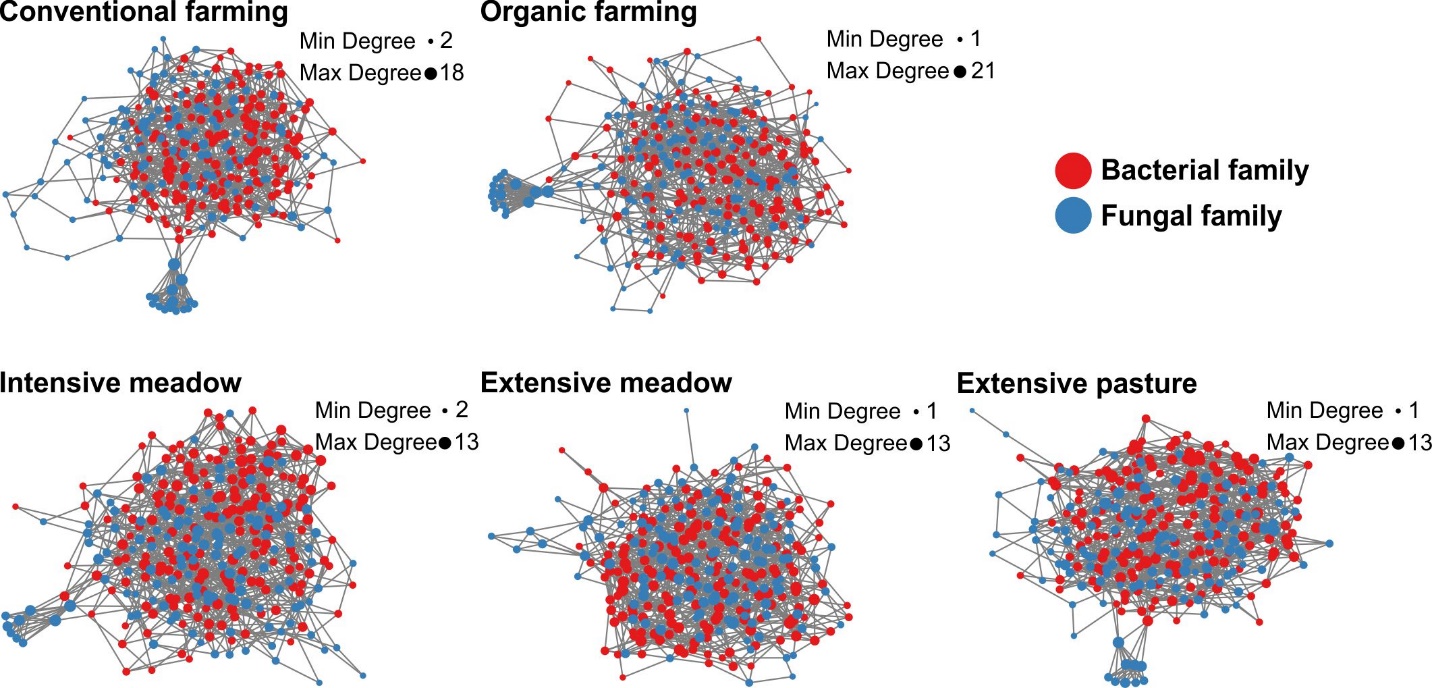


Supplementary Figure S5: Co-occurrence networks of fungal and bacterial families in different land-use types in a dry year. Each node represents one family, the size of the node represents the degree within the given network. More network properties are summarized in Tables 2, 3 and Supplementary Tables S7, S8.


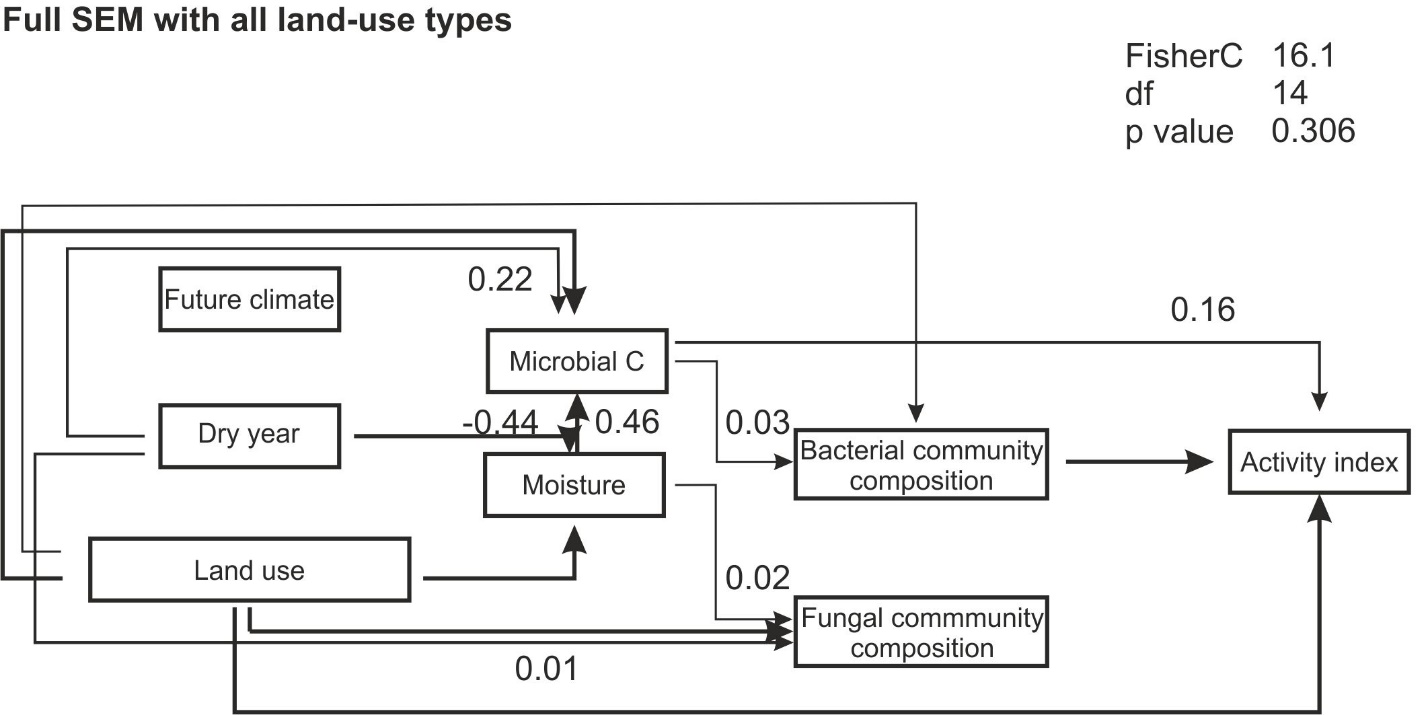


Supplementary Figure S6: Structural equation modeling of land use, climate and year effects on soil and microbial parameters. Significant paths at p = 0.05 with arrows indicating directionality and the respective path estimates are shown. Estimates for all paths, as well as land-use type specific path estimates within the SEM are summarized in Supplementary Table S9.
